# Pulmonary Edema: Consider an Unusual Suspect

**DOI:** 10.5334/jbsr.1819

**Published:** 2019-05-15

**Authors:** Charline Jopart, Philippe Hainaut, Benoit Ghaye

**Affiliations:** 1 CUSL, BE

**Keywords:** pulmonary edema, pulmonary embolism, rare complication, emergency, chest CT angiography

A 58-year-old man was admitted to the emergency department for the sudden onset of dyspnea and syncope. His medical history included a non-Hodgkin lymphoma diagnosed three months earlier; the last course of “R-CHOP” chemotherapy scheme had been administered three weeks earlier. On examination, regular tachycardia was observed at 147 beats per minute, and blood pressure was 126/95 mmHg. Arterial blood gas values were measured at room air: pH 7.41, pO_2_ 72 mmHg, pCO_2_ 33 mmHg. C-reactive protein was slightly raised at 16 mg/L, D-dimers peaked at 12000 ng/ml (normal <500 ng/ml) and T-troponin at 588 pg/ml (normal <20 pg/mL).

Chest radiography demonstrated a pulmonary infiltrate localized in the left upper lobe (Figure 1, black arrows). Transthoracic echocardiography showed right cavities enlargement and ventricular dysfunction; systolic arterial pulmonary pressure was estimated at >38 mmHg. Pulmonary embolism (PE) was strongly suspected.

**Figure 1:**
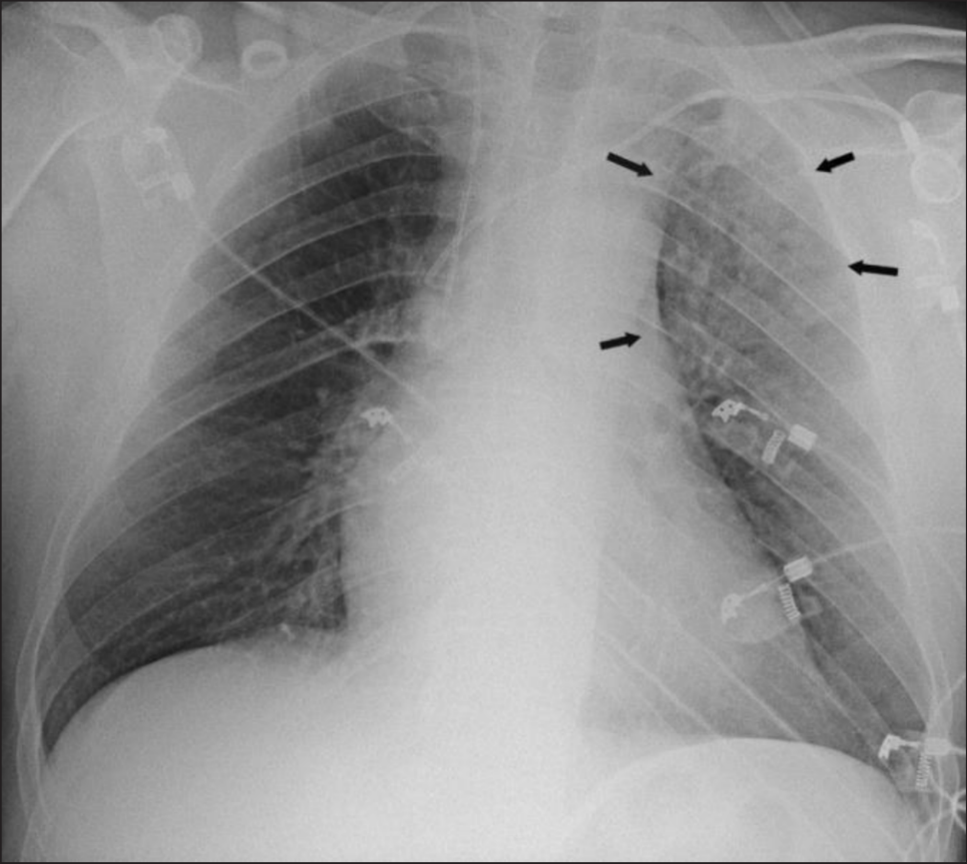


Chest CT angiography (Figure 2) revealed central ground glass opacities in the left upper lobe (black arrowheads), suggestive of pulmonary edema, and a central pulmonary embolism with a saddle clot on main pulmonary artery bifurcation and mostly occlusive thrombi in all lobar and segmental pulmonary arteries except in the left superior lobe arteries (black asterisks). After initial treatment with unfractionated heparin, he was discharged eight days later, with prolonged oral anticoagulant therapy.

**Figure 2:**
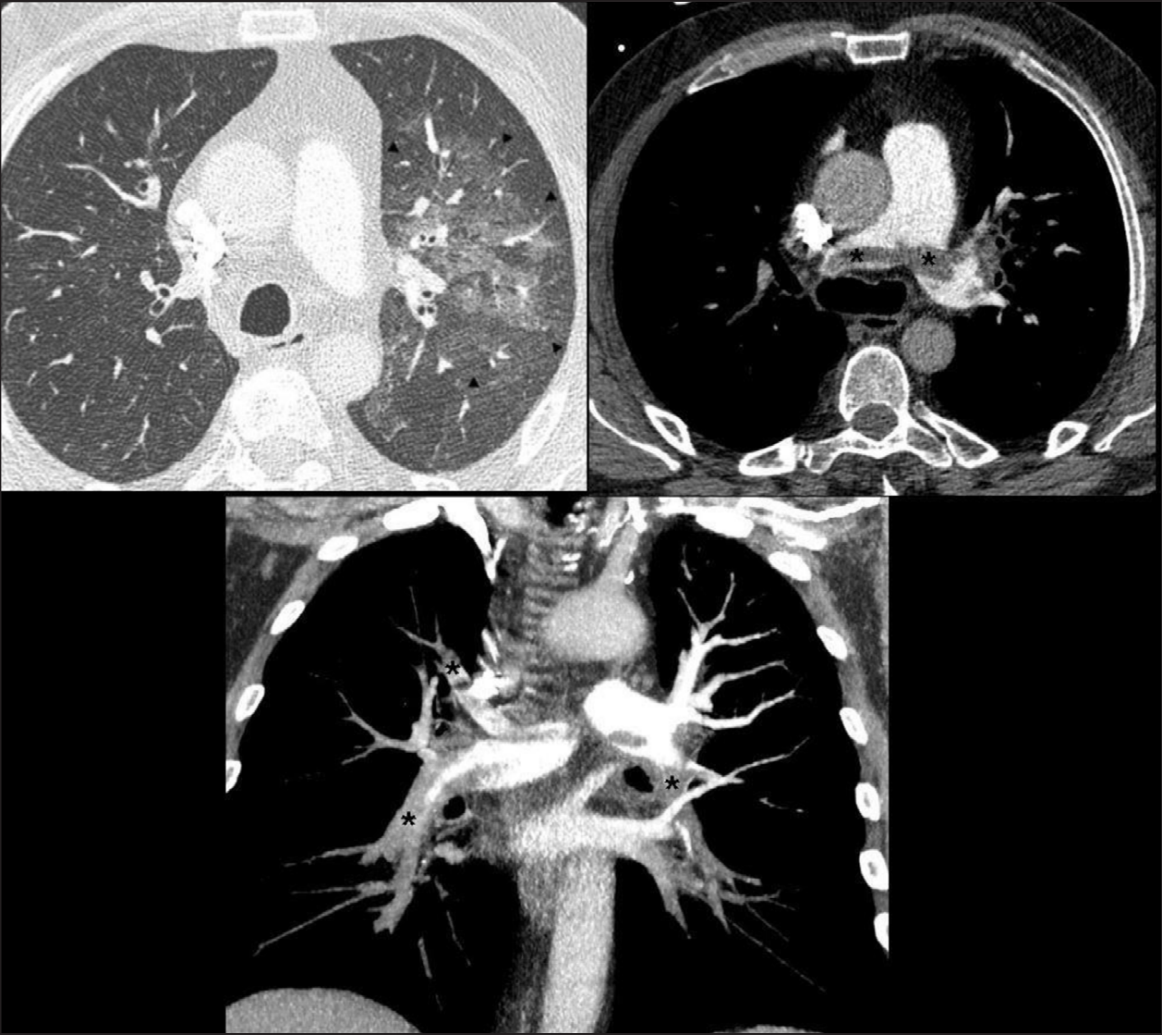


## Comment

Focal or diffuse pulmonary edema is an uncommon complication of pulmonary embolism. The pathophysiology is still unclear. It could be related to left heart failure, “hyper-perfusion” (increase in pressure and flow), explaining its occurrence only in a pulmonary area spared by the embolism, or to the release of mediators of inflammation explaining edema in the hypoperfused lobes 1. Lastly, diffuse pulmonary edema associated with focal PE has also been reported.

Pulmonary edema secondary to pulmonary embolism may mimic cardiogenic pulmonary edema. Enhanced chest CT is required for the diagnosis and should be considered when unenhanced imaging demonstrates pulmonary edema in patients with no history or clinical evidence of left heart failure and at risk of venous thromboembolism.
